# A Unifying Hypothesis for the Genome Dynamics Proposed to Underlie Neuropsychiatric Phenotypes

**DOI:** 10.3390/genes15040471

**Published:** 2024-04-08

**Authors:** George Sebastian Gericke

**Affiliations:** Faculty of Health Sciences, Prinshof Campus, University of Pretoria, Gezina 0031, South Africa; george.gericke@up.ac.za

**Keywords:** common fragile sites, stress, mobile elements, immune, RAG 1/2, genome plasticity, neuropsychiatric, GWAS, epigenetic

## Abstract

The sheer number of gene variants and the extent of the observed clinical and molecular heterogeneity recorded in neuropsychiatric disorders (NPDs) could be due to the magnified downstream effects initiated by a smaller group of genomic higher-order alterations in response to endogenous or environmental stress. Chromosomal common fragile sites (CFS) are functionally linked with microRNAs, gene copy number variants (CNVs), sub-microscopic deletions and duplications of DNA, rare single-nucleotide variants (SNVs/SNPs), and small insertions/deletions (indels), as well as chromosomal translocations, gene duplications, altered methylation, microRNA and L1 transposon activity, and 3-D chromosomal topology characteristics. These genomic structural features have been linked with various NPDs in mostly isolated reports and have usually only been viewed as areas harboring potential candidate genes of interest. The suggestion to use a higher level entry point (the ‘fragilome’ and associated features) activated by a central mechanism (‘stress’) for studying NPD genetics has the potential to unify the existing vast number of different observations in this field. This approach may explain the continuum of gene findings distributed between affected and unaffected individuals, the clustering of NPD phenotypes and overlapping comorbidities, the extensive clinical and molecular heterogeneity, and the association with certain other medical disorders.

## 1. The Search for Neuropsychiatric Disorder (NPD) Causative Genes

Initial attempts to identify major genes underlying neuropsychiatric disorders (NPDs) through linkage and association studies and mapping of candidate genes at sites of chromosomal structural alterations which were mostly observed in isolated case reports were mostly unsuccessful, and such findings were nonreplicable in many instances. The early successes obtained with mapping single-gene Mendelian disorders were gradually replaced by a realization of the need for different approaches when dealing with complex multifactorial disorders. Familial and population-based genetic studies increasingly indicated that human NPDs are polygenic, and the results of genetic studies were challenging to interpret. This led to the adoption of genome-wide association studies (GWASs) including copy number variation (CNV) studies, whole exome sequencing (WES), and whole genome sequencing (WGS) approaches.

An unexpectedly high yield of NPD risk variants was revealed by these methods and associations between more than 400,000 common genetic variants and hundreds of human traits and indirectly related disorders were identified by means of GWASs by 2023 [1]. These increasing numbers necessitated large-scale collaborative efforts, such as those coordinated by iPSYCH [2] and the Psychiatric Genomics Consortium (PGC) [3] with the latter “*harnessing the power of 800+ international scientists and 900,000 participants*”, utilizing fifteen working groups (at the end of 2023), as well as genomic diversity working groups from Africa, Latin America, and India. The trade-off when using such a huge collaborative effort is that the clinical diagnostic standardization required to prevent the introduction of site-based effects becomes very challenging.

GWASs typically report the clustering phenomenon of trait-associated genomic loci due to linkage disequilibrium, where the phenotype may be influenced by gene variants nearby. Due to gene conversion events interfering with the interpretation of linkage disequilibrium involving CNVs, the impact of CNVs on inherited human disorders is a challenging analytic problem [4]. A further major caveat is that, after the initial focus on the protein-coding regions of the genome, it has since been found that most of the common genetic variations contributing to psychiatric disorders have been found in non-protein coding regions spanning multiple genes. In this regard, it has been predicted that a significant effort will be required for the genome-wide functional annotation of these regulatory region networks [5]. This will furthermore require an understanding of that part of the noncoding human genome that is under purifying selection, likely harboring essential functional regulatory information [6], as well as a much-improved knowledge of the evolutionary forces that shape transcriptional networks. 

## 2. The Phenotype Problem in NPDs

The challenge to define ‘pure’ phenotypes represents a major problem in neuropsychiatric GWAS research. Unidentified heterogeneity is a key feature of NPDs, which detracts from large GWAS efforts to identify major causative genes/variants in common multifactorial disorders. An example of heterogeneity is represented by the presence of “hidden” endophenotypes, e.g., the evolution of different immunophenotypes with differing inflammatory and clinical profiles stratifying patients with major mood and psychotic disorders into subgroups based on HERV-W envelope protein antigenemia and cytokine profiles [7]. 

Another major problem in NPD clinical phenotypic delineation is presented by the overlapping/clustering of psychiatric phenotypes. The Cross-Disorder Group of the Psychiatric Genomics Consortium, 23andMe Research Team, the Psychosis Endophenotypes International Consortium, and the Wellcome Case-Control Consortium performed a GWAS analysis of eight psychiatric disorders and analyzed 6.8 million SNPs from nearly 233,000 individuals, along with more than 494,000 unaffected control individuals. These research subjects were enrolled for prior GWAS of schizophrenia, bipolar disorder, major depression, attention deficit hyperactive disorder, autism spectrum disorder, obsessive-compulsive disorder, anorexia nervosa, and Tourette syndrome. Nearly 150 independent genetic risk loci were identified which included 35 loci not previously linked to these conditions. Three broad groups were identified, which clustered genetically (Cross-Disorder Group of the Psychiatric Genomics Consortium, 2019) [8]. Using the same analytical approach, another GWAS analysis of eleven psychiatric disorders revealed the presence of four highly correlated groups of disorders [9]. 

Additional clinical complexity is encountered in those areas where psychiatric disorders share genetic influences with a range of normal traits and diseases, including brain structures. Relatives of probands with a psychiatric disorder also have an increased risk of developing other psychiatric disorders, which indicates that the familial risk of mental illness transcends diagnostic categories [9] (Brainstorm Consortium, 2018) [10]. While every individual is considered to harbor genetic risks for any psychiatric disorder, there is also overlap with genetic variation in traits such as general intelligence, educational attainment, subjective well-being, and sleep patterns, as well as mental health profiles in healthy individuals [1]. 

In the absence of rigorous qualitative clinical measures, individuals included in GWAS studies are usually classified as either affected or unaffected according to DSM criteria. This creates a binary categorical variable with a risk of measurement error. Allowance to accommodate a clinical spectrum of involvement leads to different problems, as definitions used for neurobehavioral endophenotypes (e.g., obsessive disorder ‘possible’ versus obsessive disorder ‘probable’) do not necessarily correlate with obtained genotypic data in any form. Attempts to circumvent the problem by gene-set clustering analytic methods often meet with variable success rates. As outlined by Kapur et al., as long ago as 2012 [11], the aim to achieve the clinical utility of diagnostic genetic testing may require a different approach: rather than comparing prototypic patients and ‘healthy’ controls, the focus should rather be on “*identifying biologically homogeneous subtypes that cut across phenotypic diagnosis*”. An idea was expressed that psychiatric diagnostic operationalized criteria may eventually have to be based on a mix of symptoms, signs, course of illness, and specific biological findings similar to those seen, for instance, in rheumatology [12].

## 3. NPD Phenotypic Complexity and Overlap between NPDs and Comorbid Disorders Complicate Genomic Data Analytics

Hypothesis-free studies designed to resolve the debate of whether psychiatric disorders are caused by a large number of common genetic variants of small effect versus multiple rare variants of strong effect indicated that common variation had a 14–28 times greater impact on schizophrenia risk than rare exonic variation or rare CNVs [1]. Other genome-wide approaches include a study of brain gene expression in different brain regions, DNA–DNA looping, and epigenomics. One of the several NIMH-supported studies conducting functional genomic studies on brain tissue from individuals with severe psychiatric disorders to assist with this endeavor was performed by the PsychENCODE consortium [13]. 

The Zhangjiang International Brain Biobank collects matched genomic, transcriptomic, metabolic, and neuroimaging data for six brain disorder cohorts and utilizes an international team of scientists from biology, medicine, computer science, physics, statistics, and mathematics to mine the accumulated data. This understandably includes gut microbiomes since the human gut has more than 1000 types of bacteria, which have been proven to be associated with brain health [14]. 

By conducting and analyzing GWASs of 13 different neuroimaging modalities both globally and across 180 cortical regions from 2347 GWASs for 2334 regional cortical brain phenotypes in 36,663 individuals from the UK Biobank (UKB) and the Adolescent Brain Cognitive Development (ABCD) cohorts, insights were gained into the genetic organization and development of the human cortex [15]. While adding another valuable step in the slow road to understanding the human brain, it is still not yet feasible to apply such information to supply diagnostically useful NPD biomarkers. The quoted studies only reflect some of the representative examples providing an idea of the major areas of pursuit and approaches utilized. While important associations between genomic, transcriptomic, metabolic, and neuroimaging data have already been demonstrated, their shared causal relationships have not been explained, challenging the abilities to solve complex, multidimensional problems in the era of biomedical big data.

Despite the amount of available information, it is still impossible to correlate clinical criteria for psychiatric disorders with genetically defined disease entities. “*A disappointing feature is that the independent significant genetic sequenced variants currently still only explain less than 10% of the SNP-heritability of schizophrenia, indicating that most of its variant architecture remains to be identified*” [1]. 

## 4. Has the Time Arrived for a New Research Paradigm?

It remains an enigma that tens of thousands of common genetic variants may influence each psychiatric disorder. Surely they do not all act on their own? How many of these alterations are present in an individual with an NPD? Utilizing fast-developing areas such as artificial intelligence to analyze hidden data patterns in data-driven mining, data noise reduction techniques, and meta-analyses, it is hoped that further insights can be gained by such additional fundamental research [14]. Major challenges remain, however, regarding the extreme complexity and high levels of disease heterogeneity associated with the low penetrance of specific gene mutations, multiple genetic–epigenetic and environmental interactions, and the influence of stochastic evolutionary processes which render most individual molecular mechanisms less than useful for clinical prediction [14]. It has been suggested that there might be no clear causative relationships at the molecular level within a complex biosystem and it is likely that stochastic genome alterations have a random probability distribution or pattern that may be analyzed statistically, but may not be predicted precisely [16,17]. 

The current scenario concerning NPDs seems to be characterized by the absence of *a priori* hypotheses, as the agnostic inductive approach of GWAS research seems to rely on an expectation of cohesive hypotheses to emerge after much expanded further data accumulation, i.e., “*in the coming years, ever-larger studies incorporating DNA sequencing, environmental exposures, and phenome-wide analyses will facilitate a more granular understanding of the genetic etiology and phenotypic spectrum of mental illness*” [8]. 

Karl Popper, in *‘The Logic of Scientific Discovery’* [18] expressed himself as a major critic of inductivism, the approach that allows the drawing of general conclusions following specific observations or patterns. He considered science to progress significantly only when an *a priori* theory is shown to be false if ongoing generated information does not fit the theory (falsification), and a new theory is introduced that better explains the phenomena. While emerging AI capabilities are expected to change the face of research and have the potential to relegate the type of research philosophy outlined here to the history of science, there may still be some merit to considering testable “bigger picture” hypotheses that are falsifiable, as a parallel approach to large-scale variant identification.

## 5. Cytogenomics of Chromosomal ‘Fragile Sites’: Can a Top-Down Approach Offer Better Options to Find More Central/Higher Levels of Involvement in NPDs?

Can an overarching model along the lines of Heng’s genome architecture theory (GAT) be proposed that ties together the disparate pieces of information? Herein, it is posited that *DNA sequences* and the *genome* represent different levels of coding and control and that the *genome* represents a more appropriate level of investigation [16]. This concept calls for a departure from gene-centric genomic research and the need to study interactions across different genetic and epigenetic domains. In this way, it is proposed that “a *lower level of seemingly infinite complexity can be converted into a higher level of simplicity*” [16,17]. In addition to considering the level of maximal effect with minimal input on the genome level, it may be worthwhile to consider that information stored in the genome determines the *potential activities* that the cells can perform, while the *actual activities* are determined by the control of the epigenetic system making use of the ‘cards’ (gene variants) it has been dealt. 

Could it be that sequencing hundreds of thousands of individuals with NPDs perhaps enlightens us about ‘potential’ based on vast genomic reserves with great intrinsic redundancy that have collectively been available to be utilized during NPD challenges? This may mean that we are still none the wiser about the mechanisms involved with the more circumscribed actual genomic activities occurring in the individual genome in distress against the background of its neurodevelopmental history. Could there be a near infinite number of ‘lottery-equivalent’ downstream gene variant combinations possible based on quite a small actual number of higher level epigenetic and genetic changes associated with the individual’s NPD, such that each person carries their pathogenic genome signature in this regard? Furthermore, the influence of different gene-level alterations that can lead to the same disease, i.e., a certain neurodevelopmental redundancy may reduce the importance of any individual gene.

**Figure 1:**
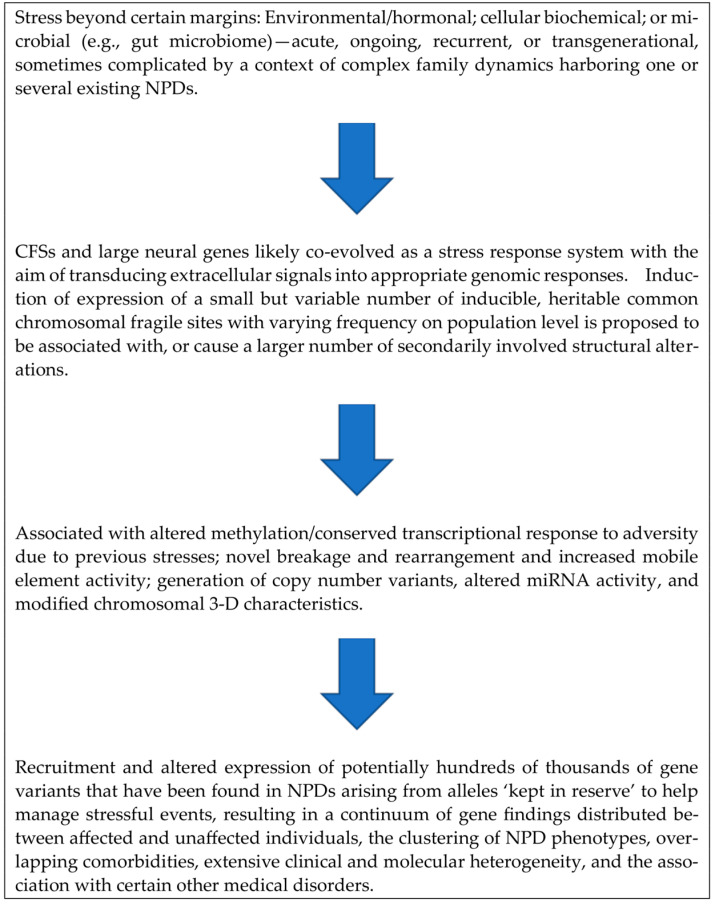
Proposed pathway of stress showing a cumulative genomic impact as it proceeds through genomic layers from higher-order common chromosomal fragile sites to downstream individual gene variant modifications.

The GAT theory underscores the importance of clinical cytogenetics, as karyotype dynamics are considered to play a central role in information-based genomics and genome-based macroevolution: “*Future clinical cytogenetics should profile chromosome instability-mediated somatic evolution, as well as the degree of non-clonal chromosomal aberrations that monitor the genomic system’s stress response*” [16,17]. This statement is in support of the main concept proposed in the current paper which is summarized in Figure 1.

## 6. Chromosomal Fragile Sites and Associated Widespread Genomic and Clinical Phenomena Have Been Associated with NPDs

The research model proposed in this article indicates the highly individualized nature of the genetic neurodevelopmental pathways operating on the whole organismal level, as well as subpopulations of neural cells. These are brought about through dynamic genomic mechanisms harnessing the incredibly varied neurodevelopmental and adverse stress repertoire of probably hundreds of thousands of alternative gene variants in a near-infinite number of combinations. Multiple avenues of genome stability research confirmed observations that certain regions of the genome are inherently more prone to breakage: so-called genome instability hotspots. Across species and kingdoms, stress-response upregulation includes the formation of DSBs or error-prone DNA polymerases within these hotspots, creating greater diversity upon non-mutagenic imperfect repair. Within these regions, one finds the common fragile sites (CFSs) that are present, or inducible, in all individuals and which seem to be conserved among vertebrates [19]. Note that this is distinct from the well-defined chromosome breakage syndromes where the underlying defect is the inability to repair a particular type of DNA damage.

It has been shown that *under normal circumstances*, controlled breakage fundamentally prevents, rather than promotes, genome instability [20]. Sequence analysis showed that at certain CFSs, fork pausing under replicative stress is located within regions of increased genetic variation in healthy human populations that could be attributed to Pol eta activity. Recent research unveiled a role for Pol eta in overcoming replication stress, reducing DNA breakage, and promoting genetic variation at CFSs [21]. A clear distinction must therefore be made between the roles of breakage and rearrangement during evolution and individual neurodevelopment, as opposed to the pathogenic induction of genomic instability associated with NPDs, neurodegenerative disorders, and cancer. Various genomic structural alterations as well as large-scale brain mosaicism occur as part of both normal evolutionary and individual neurodevelopmental mechanisms. 

Endogenous DNA double-strand breaks (DSBs) in neural cells have been implicated in the pathogenesis of neurodevelopmental disorders (NDDs). The genomic instability angle ties in with an increasing interest in neuropsychiatric genetics to understand NDDs with an early onset, including autism, ADHD, learning disability, schizophrenia, and bipolar disorder [22]. NDD risk genes were found to harbor significantly more DSBs in comparison to other protein-coding genes in neural cells and contain (gene) copy number variation (CNV) hotspots that correspond to CFSs. CNVs are linked to more than 20 neurodevelopmental or neurodegenerative diseases, as well as complex conditions such as autism, schizophrenia, and epilepsy. DNA DSB repair also appears to create complex copy-number variants [23]. Genomic fragility during human neural cell fate determination is concentrated in promoter areas and transcriptionally active genes, around chromatin loop anchors, and at the borders of topologically associating domains, which are all CFS-associated features [24]. 

All of the CFS-associated genomic structural alterations described below have been linked with NPD expression at some stage. While rare fragile sites have also been linked with neurological and neuropsychiatric disorders, this article specifically deals with CFSs which mostly replicate late in the cell cycle and frequently overlap with very long (>300 kbp) neural genes, with many that span over one megabase. Such genes take longer than one cell cycle to transcribe during which the formation of R-loops, chromosomal fragile sites, and recurrent deletions arise [25]. Thus, CFS as a central mechanism acting via a considerable number of linked structural genomic elements may represent a useful vantage point from which to appraise the complex genetics of NPDs. These genetic elements which are discussed at various points in this document include SNPs, various mutational variants, structural variants (microdeletions and duplications) and copy number variants (CNVs), an association with miRNA-rich regions/specific miRNAs, altered methylation, and findings of association with chromosomal reciprocal translocations, inversions, translocations, and chromosome fusion, as well as chromosomal instability/breakage, mobile element activity, viral integrating processes, and somatic brain mosaicism. Heritable and environmentally induced chromosomal fragile sites represent the genomic sites of a dynamic genome-wide network of altered gene expression/gene rearrangement factors; immunological; chromosomal/translocations/copy number variations, gene duplications, and LINE 1 transpositions originating at induced fragile regions associated with altered genome methylation and microRNA activity; with occasional loss of adjacent tumor suppressor genes leading to malignancy. 

The association between CFSs, cancer breakpoints, and related gene expression may be due to the close relationship between CFS and p53 tumor suppressor genes—when breaks at CFSs are not repaired accurately, this can lead to deletions by which cells acquire a growth advantage because of a loss of tumor suppressor activities. The p53 tumor suppressor protein belongs to a family of proteins that function together to modulate gene expression in response to an array of stress signals and CFS-expression-related, and in this regard, LTR class I endogenous retrovirus (ERV) retroelements were found to impact considerably the transcriptional network of p53 [26]. Neuroinflammation which activates innate immune responses through microglia is an important pathological feature of central nervous system disorders. In response to extrinsic signals such as reactive oxygen species activation, p53 coordinates microglial activation and induces the expression of microRNAs which promote a pro-inflammatory activation with secretion of inflammatory cytokines [27]. miRNA genes are frequently located at CFSs [28]. Most of the schizophrenia-associated single nucleotide polymorphisms (SNPs) are found in non-coding regions, which functionally implicates miRNAs in the development of schizophrenia. MicroRNAs are dysregulated in psychiatric disorders through both genetic and environmental influences [29]. 

One explanation for how the extensive neuronal diversity in the human brain could be achieved with only ~30,000 genes is based on an analogy with the immune system, where extensive cellular diversity is attained through immune-like somatic DNA rearrangement following chromosomal breakage [30]. A network of eighteen investigative teams representing fifteen institutions, the Brain Somatic Mosaicism Network (BSMN) supported by The National Institute of Mental Health (NIMH) links with the PsychENCODE project and the CommonMind Consortium [30]. Their research efforts indicate that each neuron may harbor hundreds of somatic mutations involving single-nucleotide variants (SNVs), small insertion/deletion (indel) mutations, structural variants including CNVs, inversions, translocations, and whole-chromosome gains or losses, as well as mobile genetic element insertions [31].

## 7. Early Observations of Chromosomal Fragility Observations Concerning NPDs

In a pioneering study in 50 schizophrenic males, during an analysis of rare chromosomal fragile sites, Garofalo found that chromosomes from schizophrenic patients displayed greater fragility than those of normal controls [32,33].

In 1995, our research group at the University of Pretoria reported a small set of CFSs that could reliably distinguish between Tourette and non-Tourette individuals [34], and in a subsequent publication in 1996 [35] we speculated whether associated (variably) expressed FS could underlie endophenotypes which would explain (a genetic basis for) comorbidity in NPDs. In an evo-devo view, in 1995, Gericke proposed that an ‘anthropogenetic’ view of behavioral alteration may assist with the elucidation of genetic changes underlying neurobehavioral variation [36].

Simonic and Gericke [37] proposed the concept that CFSs are associated with altered transcriptional regulation, such that CFS involvement could result in the production of variable and complex phenotypes.

In 1997, Simonic et al. published an observation about specific subsets of CFSs in Rett syndrome [38]. In addition, the co-occurrence of trisomy X and de novo pericentromeric inversion on chromosome 2 were found in these reported Rett syndrome patients. The cytogenetic findings at the time suggested that both X-linked and autosomal regulatory region(s) could be part of Rett syndrome. This antedated the finding in 1999, when NICHD-supported scientists discovered that most classic Rett syndrome cases are caused by a mutation within the methylcytosine-binding protein 2 (MECP2) gene on the X chromosome, with the view emerging that disruption of the RTT gene alters the normal developmental expression of various other genes, some of which must account for the neurologic phenotypes associated with this disorder [39]. The involvement of MeCP2 in methylation-specific transcriptional repression suggested the likelihood of more widespread, variable dysregulated gene expression of both X-linked and autosomal genes. These features may indicate that the genomic features underlying the clinical expression of disorders in the Rett phenotype spectrum may well represent a prototypical demonstration of the role of variable methylation and genomic instability in a broader context when studying genetic mechanisms in NPDs.

In 1998, Chen et al. reported that schizophrenia is linked to several fragile sites, some of which are unique to the disorder [40]. In 2003, Nguyen et al. reported a link between somatic mutations (genomic instability), fragile sites, and schizophrenia [41]. Their results obtained from a study in disease discordant monozygotic twins showed that a high somatic mutation rate was associated with schizophrenia.

In 2006, Gericke suggested that the observation of subsets of fragile sites expressed in certain NPDs may correlate with specific ‘chromatin endophenotypes’ and associated clinical features [42].

The Yurov et al. (2007) finding [43] of the presence of high percentages of rearranged and aneuploid chromosomes in brain cells, suggested an unexpected link between developmental chromosomal instability and brain genome diversity. This stimulated a view expressed by Gericke in 2008 [44] that CFSs may be a key features of epigenetically modified neuroplasticity. Similar to recombinase activation gene RAG-1 directed V(D)J recombination affecting specific recognition sequences which allows the immune system to encode memories of a vast array of antigens, certain CFSs have become known to function as signals for RAG complex targets. The information that RAG-1 is transcribed in the central nervous system raised the consideration that immunoglobulin-like somatic DNA recombination could be involved in recognition and (supply a structural basis for) memory processes in brain development and function as an exaptation. Cognitive-stress-induced somatic hypermutation in neurons, similar to what happens after antigenic challenge in lymphocytes, could underlie a massive increase in the synthesis of novel macromolecules to function as coded information bits that can be selected for memory storage.

The report by Yurov et al. in 2007 [43] that aneuploidy/polyploidy in human fetal tissues can be studied by advanced molecular–cytogenetic techniques at the single-cell level showed that the human developing brain has a mosaic nature, with an overall percentage of aneuploidy of about 30–35 percent. Furthermore, it was reported that mosaic aneuploidy can be exclusively confined to the brain. A following genetic study of the level of mosaic genome variations in cells of the brain autopsy tissues in healthy controls and schizophrenia revealed a three-fold increase in aneuploidy frequency in the brain in schizophrenia. It was suggested that mosaic aneuploidy, as a significant biological marker of genomic instability, could be associated with the altered functional activity of neural cells and neural networks in schizophrenia [45].

Several other similar observations also suggested the presence of somatic mosaicism both in neurotypical human brains and in the context of complex NPDs. As a result, the National Institute of Mental Health (NIMH)-supported Brain Somatic Mosaicism Network was created to study mosaicism both in neurotypical human brains and in the context of complex neuropsychiatric disorders. It was by this time accepted that many components of the DNA “damage response” are essential for neurodevelopment and that altered DNA repair could lead to somatic variation among neurons [31].

In a 2010 publication [46], Smith et al. discussed increasing evidence linking genomic and epigenomic instability to neuropsychiatric diseases including schizophrenia and autism. Data in this paper were obtained from the National Institute of Health’s database linking specific genes to schizophrenia and a PubMed search using the keywords “gene AND schizophrenia”.

Iourov et al. (2019) [47] also found that, in the schizophrenia brain, brain-specific CNVs and mosaic aneuploidy/chromosome instability were three-fold higher in schizophrenia patients than in controls.

## 8. Further Recent Developments Concerning the Various Components Associated with the Genome Instability Phenomenon

Recent improvements in sequencing-based technologies have enabled the profiling of genome-wide DNA double-strand breaks (DSBs). These ‘breakomes’ (or ‘fragilomes’) specifically map instability hotspots [48]. Among the numerous novel questions that arose during the breakome sequencing studies was whether a discrepancy exists between DNA DSBs themselves and the mutagenic events that ultimately influence disease onset and progression. Biocomputational modeling revealed several instances of mismatches occurring between predicted DSBs and structural variant densities, for instance, in tumor material. An example of the indirect relationship between a fragile site, or to put it more accurately, a *fragile region*, and disease-associated surrounding areas is provided by the first CFS to be cloned and characterized (FRA3B). Instability within this region extends for over 4.0 Mbs and the FHIT gene spanning 1.5 Mbs of the genomic sequence is found in the center of this region. Despite frequent deletions and other alterations occurring within this gene in multiple tumor types, FHIT is not a traditional mutational cancer target [49,50]. Could this be due to the altered epigenetic context at such fragile loci? Can a study of CFS regions shed further light on the gene spanning noncoding regions identified in GWASs? Are some of the answers rather to be found in the characteristics of and/or defects in the DNA repair systems?

Since CFS regions involve so many other structural features of the genome, CFSs themselves may represent intermediate features in the genotype–phenotype relationships of which they form a part. In this regard, it may be necessary to additionally take into account the topological characteristics of chromosomes to identify topological characteristics and regulatory consequences brought about by their 3-D physical proximity.

## 9. CFS and the 3-D Genome—Action at a Distance

When attempting to make connections from DNA sequence variation to a cellular mechanism in the case of common exon variants, it was found that the connection to a gene is usually indirect. Although many of the risk alleles identified by GWASs affect the expression or alternative splicing of genes in their immediate vicinity, other alleles affect genes located far from the associated SNP. Transcriptional control is associated with physical contacts between target genes and the respective enhancers brought about by chromatin folding. The topologically associating domains (TADs) (the fundamental units of three-dimensional (3-D) nuclear organization), generate extensive contacts between different genomic regions [48]. Distal fragments bound by CCCTC-binding factors (CTCFs) have been found to influence transcription at distant sites [51]. Sarni et al. (2020) [52] showed that the association of CFSs with TAD boundaries elucidates the role of topological tension generated by 3-D genome organization in chromosomal fragility and genomic stability.

## 10. CFS as “Clusters of Evolvability” in an Ocean of Genomic Stability: Clustering May Be Related to Overlapping Phenotypes

It has been long understood that mutation distribution is not completely random across genomic space and in time. The human genome can be considered a mosaic comprising regions of fragility that are prone to reorganization that have been conserved in different lineages during the evolutionary process and regions that do not exhibit the same levels of evolutionary plasticity [53]. Multiple simultaneous mutations within genes or gene families appear to be found in mutation clusters [54]. Based on bacterial insights, mutagenesis in genomic subgroups (e.g., CFS regions) might be a bet-hedging strategy that, while the risk is decreased in the larger genome, some regions are allowed to explore the fitness landscape. Mutations often occur nonrandomly in genomic clusters and are limited to small cell subpopulations [55]. Several classes of DSBs join preferentially to DSBs within the same topological domain because of proximity effects caused by spatial genome organization [56].

## 11. From Structural to Functional Clustering

GWASs typically report the clustering phenomenon of trait-associated genomic loci, which are DNA regions that involve multiple genetic variants highly correlated with each other due to linkage disequilibrium. Both CFSs as well as psychiatric disorders are found in clusters. A major question that requires further investigation is whether clustered psychiatric disorders with their overlapping comorbidities and clustering of fragile sites represent interrelated phenomena. Distinct rare and common fragile sites have been found to cluster together, appearing either on the same or on neighboring metaphase chromosome regions. An approach used to identify genomic regions harboring DSBs in neural stem cell progenitors showed that long neural genes harbor recurrent DNA break clusters. Almost 90 percent of identified clustered genes were shown to be involved in synapse function and/or neural cell adhesion and had been linked with mental disorders [57].

Clustered fragile sites and their chromosomal locations were cataloged by Mirceta et al. in 2022 [58].

## 12. Chromosomal Fragility as an Environmental Stress Related Evolutionary and Neurodevelopmental Mechanism Reshaping the Genome May Provide a Cohesive Research Context

*“I believe there is little reason to question the presence of innate systems that are able to restructure a genome. It is now necessary to learn of these systems and to determine why many of them are quiescent and remain so over very long periods of time only to be triggered into action by forms of stress, the consequences of which vary according to the nature of the challenge to be met”*.

Barbara Mc Clintock, (1978), as cited in (Jorgensen, 2004) [59]

American scientist Barbara McClintock was awarded the Nobel Prize for Physiology or Medicine in 1983 based on observations made during her studies on maize in the 1940s. Her research suggested that transposable elements (TEs) are normal components of eukaryotic genomes that have a crucial role in the shaping and evolution of vertebrates’ genomes. McClintock (1978) proposed that cells could adapt through upregulated beneficial genomic instability when sensing stress [60], but this response predictably had to be restricted within certain limits [61]. Genome-scale studies have now shown the key role of TEs in genome function, chromosome evolution, and speciation, Recent research has shown that a substantial proportion of non-exonic elements unique to mammals arose from mobile elements and the idea of numerous promoters and enhancers originating through exaptation has become accepted [62]. Again, there may be links with the noncoding gene regions identified by NPD GWAS studies.

There is also a direct link between transposing events as described by McClintock and CFS induction; by forming hairpins on flanking DNA and generating DNA double-strand breaks (DSBs) at their TE ends, DNA transposons can move between genomic sites [63]. Although most transposable elements have been rendered inactive through mutation, long interspersed element-1 (LINE-1 or L1) retrotransposition continues to diversify human genomes [64]. LINE-1 expression causes a large number of DSBs at long neural genes due to replication stress activating the DNA damage response whereby L1 retroelement mobilization in the brain is considered to diversify neuronal cell populations [65], further lending credence to McClintock’s proposal of a ‘dynamic genome’ on this level as well.

## 13. HERVs as Transposable Elements

Human endogenous retroviruses (HERVs) comprise ~8 percent of the human genome distributed over several hundred thousand loci. Mapping studies performed by in situ hybridization show that many HERVs map on fragile sites, chromosomal breakpoints, and/or hot spots. The HERV sequences dispersed in human DNA provided an abundant source of regulatory elements that have contributed to genome evolution and have been shown to influence immune receptors, as well as the synaptic plasticity of neuroreceptors [66]. HERVs have been demonstrated to be activated during certain infections associated with a risk of developing psychiatric diseases. The W family envelope protein (HERV-W env) has been linked with several neurological and psychiatric disorders; in SARS-CoV-2 seropositive cases with psychosis spectrum disorders, HERV W ENV and cytokine expression were significantly influenced by the SARS-CoV-2 virus [67].

## 14. ‘Stress’ as the Entry Point to Elucidate a Central Mechanism with Chromosomal Fragile Sites as a Network Response Underlying the Environment–Genome Interface

Stress was described by Hans Selye as a mechanism for general adaptation and the responses were shaped by natural selection because appropriate organismal or cellular responses to stress provide a selective advantage. Despite this early emphasis on its benefits, stress as a disease-causing response tends to receive most of the attention [68]. As predicted by McClintock, mutagenesis that is upregulated by stress responses generates transient, genetic-diversity bursts that can propel evolution, which may be enhanced by the fact that major breakpoint region (MBR) hot spots fall in or near active genes. Favoring mutations to genes currently under selection might additionally accelerate evolution [69].

Conrad Waddington created the term ‘epigenetic programming’ in the 1940s when he investigated the effects of environmental stresses. Waddington proposed that, since multiple determinants work together to define ‘form’, most single determinants would not cause phenotypic variation. He speculated that environmental stress could reveal cryptic, unexpressed variation and induce a wide range of striking phenotypic changes [70].

Today, it is known that the ‘fragilome’ forms a dynamic genome network stress response which is important from both an evolutionary and neurodevelopmental (“evo-devo”) viewpoint. Based on a comparative cytogenetic study among different primate species, chromosome bands implicated in evolutionary reorganizations were identified in the karyotypes of Papionini and Cebus species. More than 80% of these evolutionary breakpoints are located in chromosome bands that express CFSs and/or contain interstitial telomeric sites (ITSs) [54]. Evolutionary chromosomal breakpoint regions are enriched in structural variants, SNPs, genes, and pseudogenes [71]. The latter may participate in gene conversion events. The two molecular mechanisms that can explain the phenomenon of gene conversion are mismatch repair and DNA gap repair synthesis. Both mechanisms can occur in the DNA double-strand break model. Somatic mosaicism resulting from interallelic gene conversion has been indicated to represent an important modifier of human inherited disease [72]. Mathematical modeling has shown that stress-inducible mutagenesis accelerates adaptation in changing environments [54] and a question that comes to mind is whether the associated selection of adapted gene variants required for a diversifying stress response is responsible in the apparent increase in autism and cancers as a tax penalty in novel hostile environments? What lies behind the high frequency of de novo findings in this regard?

How can environmental cues be relayed back to L1 retrotransposition? There exists evidence that androgenic steroids and steroid-like compounds can induce L1 activity. The cytogenetic observation of despiralized lesions, cytogenetically similar to fragile sites within specific heterochromatic (methylated) regions, was considered to indicate the importance of methylation concerning fragility at various loci [73]. It is considered likely that DNA conformational changes and novel DNA–protein interactions contribute to fragile site expression following an altered methylation background [74]. It has been argued that cortisol and sexual hormones influence global methylation which underlies the links between the stress response and mental health pathways involved in the expression of psychiatric disorders [75]. There is an increasing body of knowledge on the influence of differential DNA methylation of specific genomic regions in psychiatric disorders. Global DNA methylation levels can supply an overview of biological functioning that is regulated by cortisol and the sex hormones and which influences metabolic and environmental influences on gene expression. In humans, a “conserved transcriptional response to adversity” in circulating leukocytes has been identified. This is likely to be regulated by 5-methylcytosine which regulates gene transcription [76]. By combining hair cortisol measurements with whole-genome DNA-methylation sequencing to determine chronic stress biomarkers, it was demonstrated in a group of 5-year-old children that high cortisol associates with a genome-wide decrease in DNA methylation and SINE transposons (non-autonomous retrotransposons depending on enzymes encoded by LINE sequences) and genes important for calcium transport were targeted which are commonly affected in stress-related diseases. A zinc-finger transcription factor, ZNF263, was additionally identified. This transcription factor, whose binding sites were exceptionally frequent in regions characterized by methylation loss, was previously shown to be involved in the defense against retrotransposons [77].

## 15. Evolutionary Layering

Despite evolutionary novelty being a striking aspect of evolution, it is not accounted for in classical evolutionary theory. This is perhaps due to reliance on the classical tedious process of mutation and selection and a reticence to be enthusiastic about anything having the slightest Lamarckian flavor. Evolution builds with the tools available on top of what it has already built—much novelty consists of repurposing old functions in a different context. Mathematical and biocomputational models endorse aspects of evolutionary innovation, one example of which is a constructive novelty, where lower levels are applied as informational scaffolds to generate novel levels of biological organization [78], similar to the ideas discussed earlier according to the GAT theory [16]. 

## 16. Does Novelty Stress Drive the Evolution of Form, Function, and Cerebrodiversity?

Since McClintock’s original discovery, sufficient evidence has accumulated that transposed elements can confer stress inducibility to nearby genes or protect those genes against stress. Environmental influences such as stress, infections, nutrition, or other environmental factors that affect the mother, appear to influence L1 mobility in newborn neurons during embryonic development. Induction of L1-induced neuronal diversity could increase the neurobehavioral spectrum originating from a single genome [65]. Several gene ontologies were identified that were affected by the L1 burden. These include glutamatergic signaling and immune functions which have previously been linked with schizophrenia [79].

## 17. The Immune System, Memory Consolidation, and Traumatic Stress Memory

The evolution of an adaptive immune system in jawed vertebrates, characterized by the somatic rearrangement of T and B cells, is supported by Class II TE activity. This results in a vast repertoire of antibodies and receptors [80]. The same double-strand break mode linked with transposing events is employed by the V(D)J recombinase at signal-end/coding-end junctions during the generation of antibody diversity. A component of the immune system recombinase (RAG-1) is expressed in cortical and hippocampal NPCs during mouse neurodevelopment. Antibody production was also demonstrated in astrocytes which are increasingly linked with neurobehavioral changes and where astrocytes were found to exhibit the classic enzymatic machinery involved in V(D)J recombination [79]. Furthermore, gene conversion involving pseudogenic sequences during class switch conversion or recombination of the constant region increases the creation of a vast diversity of immunological recognition molecules from a limited number of initial gene segments [81].

*“Learning and inheritance (genetic, epigenetic, cultural) may be fused under a term of memory. Memory is essential for life, for its ability to reproduce the developmental process again and again, in different times and contexts, permeating the emergence of new phenotypes. What is crucial for our understanding of evolution are the means through which memory is passed and such vary in their nature. From learning to genetic inheritance, this all may be perceived as a manner through which life uses its experience”*.

[82]

The immune V-(D)-J breakage and recombination paradigm used for immune recognition and memory formation appears to have been adopted by the brain as evidenced by the expression of the RAG-1 gene in the hippocampus. Notably, neurons undergo DSB formation in response to various forms of neuronal stimulation including physiological neurobehavioral tasks [56]. It is tempting to extrapolate this information to the possibility that a novel ‘information bit’ reaching the brain RAG system acts much like a foreign antigen, resulting in a somatic hypermutation and selection process to be established as a lasting memory of importance for survival.

Links have been proposed to exist between locus-specific oncogenic lncRNAs, aberrant local chromatin structure, and the generation of new epigenetic memory at a fragile site [83]. Biological evolution and human cognition have been speculated to represent alternative forms of natural information processing systems. In a less stable environment, evolution appears to support the more creative method of “generating and testing”. The variability generator for evolution is the generation of novel variants [84].

## 18. What Would the Required Theoretic Assumptions Be of Coordinated Stress Responses in the Consideration of a Genomic Model to Develop a Testable Hypothesis in Psychiatric Genetic Research?

(a) Genomic mechanisms should be identifiable which should allow the assessment of diversifying genomic reorganization with linked genomic protective measures at a rate infinitely faster than the standard processes of mutation and selection all happening within an individual’s lifetime. Thus, a combined epigenetic–genetic approach to correlate fragilome-associated characteristics with NPD gene variants would be required.
(b) One such central mechanism, i.e., the ‘fragilome’, allows for chromosomal level genomic breakage and rearrangement/recombination interacting with external or internal stress, where all the structural elements described in association with NPDs can be accommodated. This chromosomal network of unstable/plasticity genomic regions can be visualized inter alia through common chromosomal fragile site expression and fragilome gene sequencing. A first step towards answering the question of a possible functional role of L1-induced genomic variation in the brain will be to comprehensively catalog and characterize new insertion sites at the single-cell level. We already know that an unexpected source of variation exists within mammalian brains that generates diversity shaped by neurodevelopmental pathway events within every individual. The elucidation of disturbed private pathways leading to NPD clinical manifestations may save a lot of cross-sectional mass-scale collections of further NPD variants.
(c) In humans with complex behavioral repertoires, mechanisms of memory storage and retrieval need to be linked with previous emotional and behavioral responses of survival value for which an integrated neuro-immuno-endocrine system is essential, with immune-like mimicking mechanisms in the brain which also allow for transgenerational effects.
(d) According to the concept of Weismann’s barrier, the germ line is protected from somatic DNA changes during an individual’s lifetime. This theory negates the Lamarckian notion that adaptive somatic mutations in an individual could be passed on to their offspring. However, if the mechanism that creates this variability has an impact on fitness, it will be subject to natural selection. Hence, assuming that somatic diversity attainment in the brain is genetic, selection would tend to maintain this genetic mechanism. This assumption can be tested by analyzing variants that show whether TEs can generate heritable epigenetic mutations. It may furthermore be useful to keep in mind the type of epigenetic germline modification observed by Waddington, as he showed that persistent environmental stress and selection for phenocopies could recover variants that were heritable even in the absence of the stress that first elicited the phenotype, i.e., the initial modification must have been ‘hardwired’ in the germline context [85]. Then, there is the controversial but fascinating theory [86] of an immunologically mediated soma-to-germline flow of information contained in cDNA retrotranscripts copied from the pre-mRNA of B lymphocytes following somatic hypermutation of rearranged V(D)J genes). In the context of NPDs, this would be especially interesting if this phenomenon could be demonstrated to involve information flow from brain-rearranged V(D)J genes to the germline, conveying crucial hippocampal survival memories established in V(D)J-associated RAG genes. All of these mechanisms would serve the purpose of transmitting and maintaining a genetic repository of critically learned survival behaviors through the germline.
(e) More comprehensive research is required about the moment a general stress response switches DSB repair from accurate to mutagenic MBR, causing small mutations and deleterious genomic rearrangements. This penalty can perhaps be viewed as a spin-off from having the advantage of a process exploring novelty but functioning ‘on the edge’. Whereas it has long been argued that non-mutagenic DNA repair could not evolve, the ability to remove the mutagenic component from MBR without reducing repair demonstrates that this is not the case [54]. Lastly, CFS studies to be included/utilized include those found in medical conditions comorbid with NPDs such as fibromyalgia and PTSD.
(f) In order to conceptually advance the study of CFSs in NPDs, it will have to be disengaged from the primary cancer oriented/genomic instability research approach and associated terminology associated with pathogenicity. This is necessary to place the emphasis on an appreciation of their other evolutionary–neurodevelopmental, non-pathological role in human biology. Elucidation of the architecture of these complex regulatory networks is one of the main challenges for the future. Initially, microarray-based techniques became available for the genome-wide mapping of in vivo protein-DNA interactions and epigenetic flags [87]. “Mocap” is a method that identifies transcription factor cell-type-specific classifiers through an integrated analytic approach [88]. Another method, MiDAS-Seq, is based on novel high-resolution sequencing which allows direct sequencing of fragile sites and other genomic regions that remain under-replicated prior to mitotic entry [89]. The heterogeneous regulatory landscape encountered in complex diseases will furthermore stand to benefit from the development of single-cell multimodal-omics technologies aided by multi-omics integration tools [90].

## 19. Conclusions

In this paper, genomic stress responses have been suggested to underlie multiple levels of structural genomic alterations associated with the expression of NPDs. The observed clinical and molecular heterogeneity is proposed to be progressively magnified by the downstream effects initiated by a smaller group of higher-order chromosomal level alterations under the control of the ‘fragilome’. Current research identifying an increasing number of NPD-associated gene variants appears to be focused on identifying the elements furthest away from the smaller number of initiating central higher-order modifications. From an evolutionary perspective, the involvement of large neural genes at CFSs, and late replication to protect such regions from potential DNA damage during vulnerable periods in the cell cycle, linked with genomic diversification when placed under stress, makes sense when one considers a default system setting to explore novelty by creating “on the edge conditions” both in genomics and resultant behaviors. Pathogenic effects ensue when this spills over into NPDs, neurodegenerative disorders, or cancer.

Is the relationship between the gut metagenome and variant expression of brain genes mediated by altered fragile genomic regions? No significant research findings are currently available on the impact of variation in the gut metagenome on the interaction with, and expression of, clinical disorders associated with common chromosomal fragile region brain genes. A significant relationship has, however, been described in a mouse model of the rare fragile-site-related fragile X syndrome [91], which is the most common form of an inherited intellectual deficit as well as representing the prime cause of monogenetic autism spectrum disorder. Future emphasis on a gut microbiome–brain fragilome angle of investigation may hold the promise of significant further insightful gains into mechanisms influencing the expression of large fragile-region-associated brain genes and NPD clinical expression.

**Design of anti-evolvability drugs for cancer—caveats for off-target neuropsychiatric effects?** The concept of the same genomic processes being involved in normal and abnormal genomic instability has certain implications when considering the design of more genomically invasive cancer treatments that are directed towards ‘evolved’ cell biological properties of cancer [92]. The development of anti-evolvability drugs as a cancer treatment strategy needs to be observed closely and, if used, to be applied with great circumspection to avoid off-target neurobehavioral epigenetic and genetic effects. This is especially important for neurobehavioral genetic variants which could become involved by proxy, as NPD genetic risks vary across dimensions that are normally distributed in the population. The proposed modulation of these processes in cancer treatment may represent a very high-risk strategy, unless we develop a better understanding of at which point the evolutionary advantageous process of diversification assumes a pathological character and what the effects would be if the latter is suppressed beyond the point of the switch from a beneficial to a pathological process.

## Data Availability

No new data were created or analyzed in this study.
